# Aging in place: Programs, challenges and opportunities for promoting healthy aging for older adults

**DOI:** 10.1002/nop2.1872

**Published:** 2023-05-28

**Authors:** Brenda Owusu, Balkys Bivins, Beatrice Remy Marseille, Diana‐Lyn Baptiste

**Affiliations:** ^1^ Director of Adult‐Gerontology Primary Care Nurse Practitioner Program University of Miami School of Nursing and Health Studies Coral Gables Florida USA; ^2^ School of Nursing and Health Studies University of Miami Coral Gables Florida USA; ^3^ Johns Hopkins University School of Nursing Baltimore Maryland USA

The aging population is rapidly growing in the United States, with estimates projecting that by 2030, one in five Americans will be over the age of 65 (U.S. Census Bureau, 2020). The state of Florida has a high proportion of older adults, with 21.3% of its population being 65 or older, second only to Maine (Population Reference Bureau, 2020). Nationally, the older population is expected to grow significantly in the future. It is estimated that by 2040, there will be about 80.8 million older persons, more than twice as many as in 2000. That number is projected to reach 94.7 million in 2060 (Administration for Community Living, 2020). In Florida, which continues to experience a surge in migration of older adults, the 65 and older age group was the fastest growing between 2010 and 2021 with an increase of 40.3% (USAFacts, 2022). Persons 65 years and older are also living longer with an average life expectancy of 19.6 years. More than half (61%) of individuals age 65 and older live with a spouse or partner and about 27% live alone, compared to a relatively small number who live in nursing homes (Administration for Community Living, 2020).

## 1 AGING IN PLACE

‘Aging in place’ is the ability to remain in one's own home and community as one age, with access to the necessary support and services to maintain quality of life (Administration on Aging, 2019). As the population continues to age, there is a growing need for programs and initiatives that support aging in place and improve the quality of life for older adults. In a 2018 study published by the American Association of Retired Persons (AARP), 86% of adults aged 65 or older reported wanting to stay in their own homes for as long as possible (AARP, 2018).

Aging in place has been linked to several health benefits, including improved quality of life, reduced healthcare costs and increased social connectedness (Centers for Disease Control [CDC], 2020). However, aging in place can be challenging for many older adults because of declining health, loss of independence and increased risk of injury. One in three older adults who experience a decline in independence may also require assistance with daily activities, such as bathing, dressing and eating (Freedman et al., 2016). A positive outcome of the aging‐in‐place programme would be that older people can get the support needed to maintain their independence while residing in their homes rather than being transferred to live in a nursing care facility. The literature supports that when older persons can age within their own residence, there is improved quality of life (van Leeuwen et al., 2019).

## 2 CAPABLE PROGRAM

To support aging in place and improve the quality of life for older adults, there is a need for programs and initiatives that address the challenges of aging, such as health issues, home modifications and access to community resources. One such program is the community aging in place—advancing better living for elders (CAPABLE) program. The CAPABLE program is a 5‐month (10‐visit) home‐based program involving a registered nurse, occupational therapist and handy worker that work with the older adult to develop goals and action plans to change behaviours to improve health, independence and safety (Szanton et al.,  2014). The registered nurse targets pain reduction, medication reconciliation and communication with the primary care provider, and assists with incontinence supplies orders. The occupational therapist teaches clients how to perform ADLs safely at home, which often includes meal preparation, bathing, dressing, strength training, home management and caregiver education to teach the technique of home safety. The handy workers provide home modifications such as the installation of grab bars in the shower or a ramp to make the house wheelchair accessible. Members acquire skills such as learning how to use new equipment and making home adjustments to increase function and safety. Participants receive tools, home modifications and learn skills and exercises to improve function and safety. CAPABLE has been implemented in 34 states in the United States, but not yet in Florida where it is, most greatly needed.

The CAPABLE program has been shown to be effective in improving the quality of life and reducing hospitalizations and nursing home admissions for older adults. Participants in the CAPABLE program experienced improvements in daily activities, home safety and quality of life. Additionally, the program has been found to be cost‐effective, with potential savings of $10,000 per participant per year in healthcare costs (Szanton et al., 2020). While the CAPABLE program has been successful in other states, its implementation in Florida faces several challenges. For example, funding for the program may be a barrier, as Florida has not yet included CAPABLE in its Medicaid waiver or Medicare Advantage supplemental benefits. Additionally, there may be a lack of awareness among older adults and healthcare professionals about the benefits of the program.

**FIGURE 1:**
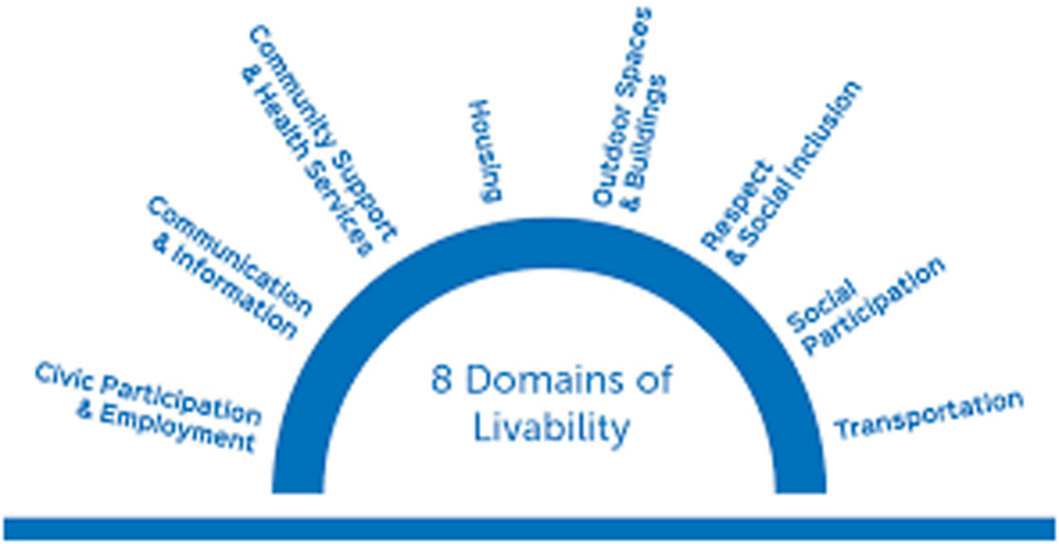
Age‐friendly community features eight domains of livability—Age‐Friendly Sarasota.

To address these challenges, policymakers and healthcare professionals need to work together to increase awareness of the CAPABLE program and secure funding for its implementation in Florida. In addition to the CAPABLE program, other initiatives and programs can support aging in place and improve the quality of life for older adults. One such initiative is the Age‐Friendly Sarasota program, which aims to make Sarasota County a livable community for people of all ages. The program focuses on eight domains of livability, including transportation, housing and social connectedness, among others (Age‐Friendly Sarasota, n.d.). The Age‐Friendly Sarasota program has been successful in improving the quality of life for older adults in the community by implementing various initiatives such as ‘Complete Streets’ policies that promote safe and accessible transportation options, affordable housing initiatives and community engagement programs that foster social connectedness (Age‐Friendly Sarasota, n.d.). The program's success has led to Sarasota County being designated as the first ‘Age‐Friendly’ county in Florida by the World Health Organization (WHO) (2015). This model serves as a strategic approach for aging in place and illustrates the eight domains of the livability of the Age‐Friendly Sarasota program (Figure 1).

## 3 IMPLICATIONS FOR NURSES

Implementing aging‐in‐place initiatives has some positive implications for nursing such as providing opportunities for nurses to work in home‐based care settings, promoting a patient‐centred approach to care and improving coordination between healthcare providers and community resources. Furthermore, in the U.S., Nurses and Adult‐Gerontology Nurse Practitioners (AGPCNP) are at the forefront of promoting successful aging in place due to their unique competencies of being advocates, health educators, change agents and case managers. AGPCNPs with expertise in older adult care are ideal persons to advocate for and actively participate in initiatives that foster aging in place.

## 4 CONCLUSION

The changing landscape and sociodemographic shift in the United States have implications for the implementation of programs such as the CAPABLE program. In Florida, specifically, the aging‐in‐place initiatives such as CAPABLE will be of great benefit due to its high older adult population. Remaining in one's own home and community as older adults age, with access to necessary support and services, can foster life satisfaction, a positive quality of life and self‐esteem, all of which are essential for maintaining health, and wellness throughout the aging process. CAPABLE focuses on the promotion of independence, safety, prevention and problem‐solving, building skills that participants can use in the future.

## FUNDING INFORMATION

None.

## CONFLICT OF INTEREST STATEMENT

Authors declare no conflicts of interest.

## ETHICS STATEMENT

None.

## Data Availability

None.
